# Simulation and Experiment on Droplet Volume for the Needle-Type Piezoelectric Jetting Dispenser

**DOI:** 10.3390/mi10090623

**Published:** 2019-09-18

**Authors:** Shizhou Lu, Xi Chen, Hai Zheng, Yupei Zhao, Yizhou Long

**Affiliations:** 1 School of Mechanical, Electrical & Information Engineering, Shandong University at Weihai, Weihai 264209, China; 2 School of Mechatronics Engineering, Harbin Institute of Technology, Harbin 150001, China

**Keywords:** droplet, droplet size, needle-type, piezoelectric actuator, microelectronics packaging field

## Abstract

The needle-type piezoelectric jetting dispenser is widely applied in the microelectronics packaging field, and it is important to control the droplet size to ensure that the droplet jetting process is successful. In this study, we analyzed the influences of system parameters, such as air pressure, nozzle size, needle strokes, and liquid properties, on droplet size and morphology by considering the droplet formation and separation process through a numerical simulation. An experimental platform was also designed to verify the reliability of the simulations and further analyze strategies for controlling the droplet size. We found that the droplet volume can be increased with an increase in air pressure, needle strokes, and nozzle size until the flow-stream or satellite droplets appear. On the other hand, very small values of these parameters will lead to adhesion or micro-dots. A large nozzle and needle displacement should be chosen for the high-viscosity liquid in order to produce normal droplets. The results also show the recommended ranges of parameter values and suitable droplet volumes for liquids with different viscosities, and these findings can be used to guide the droplet volume control process for needle-type jetting dispensers.

## 1. Introduction

Micro-droplet jetting technology is widely applied in the microelectronics packaging field to perform bonding, filling, and connecting functions [1,2,3]. In particular, with the development of semiconductor technologies, chips have become smaller and have more pins [4,5,6]. In these circumstances, adhesive droplets with a smaller size and a higher volume accuracy are required to ensure that the chip packages are of high quality.

Over the years, many kinds of droplet generation and manipulation techniques have been developed and applied widely in different research areas [7,8,9,10,11]. In the microelectronics packaging field, these methods can be divided into two types by considering the droplet dispensing process: contact and non-contact dispensing [4,12]. The contact method is suitable for dispensing high-viscosity adhesives and producing large droplets [4,13]. However, it requires a vertical motion to establish a dispensing gap between the nozzle and the circuit, which lowers the dispensing accuracy and efficiency [4,14]. The non-contact method can jet the droplet out directly without a vertical movement. Its advantages of high accuracy and a small droplet size meet the requirements for droplet ejection in the microelectronics packaging field [4,12,15,16].

In recent years, the needle-type non-contact dispensing approach, that is, the use of a positive striking needle to hit and separate the adhesive into droplets, has been widely applied in the microelectronics packaging field [11,17]. The needle is usually driven by a piezoelectric, a pneumatic, or an electromagnetic actuator [14,17]. For example, a pneumatic jet dispenser has been studied by some researchers [18,19]. This kind of dispenser utilizes an electromagnetic valve to make the needle vibrate. The jetting cycles and droplet morphology can be affected by the hysteresis of the valve and the air pressure. The giant magnetostrictive material (GMM)-based jet dispenser has also been studied in this field [20]. As an inductive loaded coil is necessary for the GMM actuator, the operating frequency of the GMM-based dispenser exceeds 250 Hz.

Due to the advantages of a fast response, an accurate output, and ease of operation, the piezoelectric dispenser has a wide range of applications in production. For example, Juncheol et al. designed a jetting dispenser system using two piezo stack actuators and studied its performance through finite element analysis and experiments [21,22,23]. Zhou et al. proposed a novel high-speed jetting dispenser driven by two piezoelectric stacks and investigated the flow velocity and volume according to a multi-physics coupling simulation model [24,25,26]. Lu et al. manufactured a rhombic amplifier-typed piezoelectric dispenser and studied the droplet jetting process and summarized the influences of system parameters on droplet formation and separation mechanism [7,27].

These studies can guide the design and parameters’ settings of the proposed needle-type dispensers to avoid some unexpected jetting status like adhesion, satellite dots, or sputtering. However, the issues of dispensing volume and droplet separation process are discussed separately in these papers. It should be noted that it is more helpful to study the droplet volume only when the successful formation and separation process of droplets is ensured.

In this paper, we further study the influences of system parameters on droplet size by considering the droplet ejection process. We also use the finite volume method to conduct a simulation. First, we introduce and analyze the configuration and working principle of a needle-type piezoelectric jetting dispenser and the droplet dispensing platform. Then, we model the dispenser using Fluent to conduct some simulations. In the next step, we use the finite volume method to study the droplet formation and separation process and the changes in droplet morphology with an increase in volume. After that, we comprehensively analyze the influences of system parameters, including air pressure, nozzle size, needle stroke, and liquid properties, on the droplet volume and morphology. Finally, an experimental platform is constructed to verify the simulations and further study strategies for controlling the droplet size. Based on the simulation and experiment results, we also provide the recommended ranges of parameter values and suitable droplet volumes for liquids with different viscosities. The results can be used to control the droplet size and morphology.

## 2. Needle-Type Jetting Method and Simulation Approach

### 2.1. Dispensing System and Working Principle

**Figure 1:**
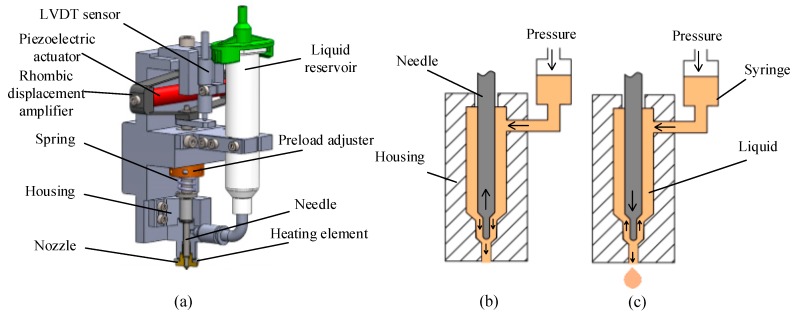
Dispenser configuration and working principle: (**a**) Configuration; (**b**) the needle’s upward movement process; (**c**) the needle’s downward movement process.

The configuration and working principle of the needle-type piezoelectric jetting dispenser are shown in Figure 1. As Figure 1a shows, the dispenser is composed of a piezoelectric actuator, a rhombic displacement amplifier, a needle, a nozzle, a liquid reservoir, a linear variable differential transformer (LVDT) sensor, and a ceramic heating element. The piezoelectric actuator is fixed horizontally on the internal part of the rhombic amplifier. Once the piezoelectric actuator generates a displacement, the rhombic amplifier will magnify it before it drives the needle to vibrate in the vertical direction. The upper part of the needle is connected to the bottom of the rhombic amplifier, and the lower part is located in the nozzle. The ceramic heating element, which is used to change the viscosity of an adhesive by controlling the temperature, is placed between the nozzle and the housing part. The liquid reservoir is used to hold the abovementioned adhesive, which will flow into the chamber of the housing part as shown in Figure 1b.

**Figure 2:**
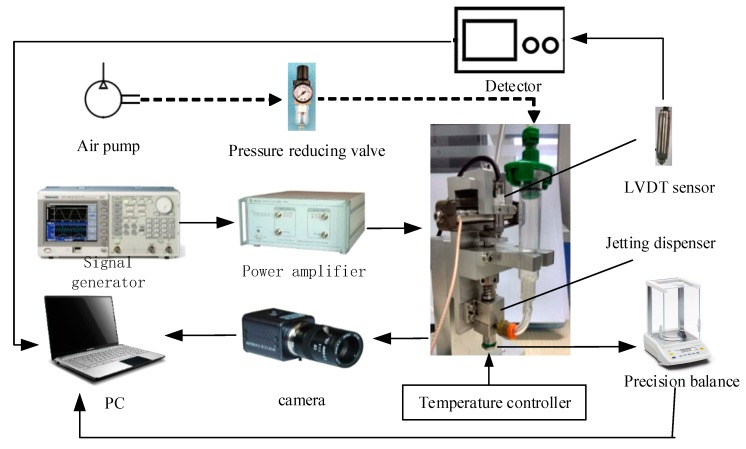
The schematic of the experimental platform.

The working principle of the dispenser can be described as follows: when the piezoelectric actuator generates a displacement, the rhombic amplifier will move the needle up to amplify the displacement in the vertical direction, and then the adhesive will flow out from the nozzle (Figure 1b). In the next step, when the piezoelectric actuator moves back to its original position, the needle will fall down quickly and hit the adhesive in the nozzle. Then, the flow will be separated into two parts. The liquid that has flowed out from the nozzle will leave the nozzle in the form of a droplet (Figure 1c). In this study, a droplet jetting and volume testing experimental platform was also constructed to undertake some experiments, as shown in Figure 2.

As Figure 2 shows, the platform consists of the needle-type dispenser, a voltage signal generator (AFG1062, Tektronix, Beaverton, OR, USA), a power amplifier (E01.02 Coremorrow, Harbin, China), an LVDT sensor (SL1500, AMETEK, Berwyn, PA, USA), a precision balance (FB224, SOPTOP, Shanghai, China), a camera (Manta G-223B, Allied Vision Technologies, Stadtroda, Germany), and an air pressure control system. The dispenser uses a piezoelectric actuator (PST150/5/60, Piezomechanik, München, Germany) to provide force and a self-designed rhombic displacement amplifier to magnify the displacement. The LVDT sensor is used to test the needle displacement during the vibration process. The signal generator and the power amplifier are used to produce and magnify the required voltage signal for the piezoelectric actuator. The camera is used to capture images of the droplet during the experiments. The air pressure control system is used to adjust the pressure during the experiments. The precision balance is used to weigh the ejected droplet, and the value is converted to the volume according to the density of the liquid. 

### 2.2. Simulation Model and Parameter Settings

In this study, we chose Fluent Software to conduct the simulation works, and the finite volume method is adopted by Fluent to solve fluid simulation problems. The finite volume method directly integrates the differential equations over the control volume, and the obtained equation has clear physical meaning. Given that the formation and separation process of a droplet is a typical gas–liquid two-phase flow problem, we selected the volume of fluid (VOF) model to capture the morphological changes in the droplet. Besides this, the flow in the nozzle was processed on axial symmetry without circumferential velocity, because a droplet shape’s is axisymmetric and the nozzle is parallel to the gravitational field and the direction of the needle’s vibration. Thus, the droplet jetting process is a two-dimensional axisymmetric problem.

**Figure 3:**
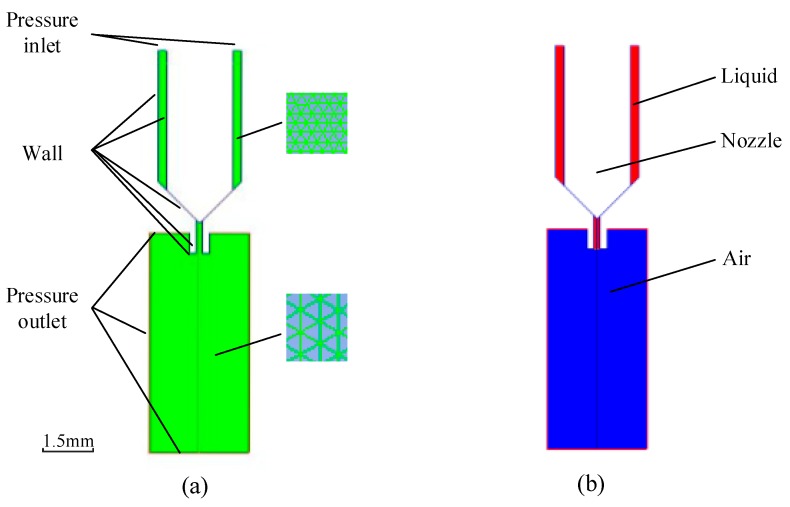
Boundary conditions of the simulation model: (**a**) Gambit mesh model; (**b**) computational fluid dynamics (CFD) simulation model.

Figure 3a shows the meshed physical model created by Gambit Software. In order to balance the accuracy and speed of simulation, the interval size of the air part was set to 0.01 mm and the liquid part was set to 0.005 mm. The triangular mesh and the pave type were provided for the model. It can be seen from the figure that the upper part of the nozzle and the gas interface were set as the pressure inlet and pressure outlet boundaries, respectively. Each wall of the nozzle and the needle utilized the wall boundary. The symmetry axis of the model used the axis boundary. In the computational fluid dynamics (CFD) model (Figure 3b), the upper part of the nozzle was set to liquid, and the areas between the nozzle and the needle were filled with liquid at the initial moment. The outside part of the nozzle was set to air, and the pressure outlet of the gas interface was set to 0 MPa. Besides this, the user-defined function (UDF) file was adopted to move the needle up and down. The vibration frequency and the displacement of the needle were controlled by the UDF file. Table 1 shows the ranges of parameter values that were used during the simulation.

## 3. Droplet Formation and Separation Process

**Figure 4:**
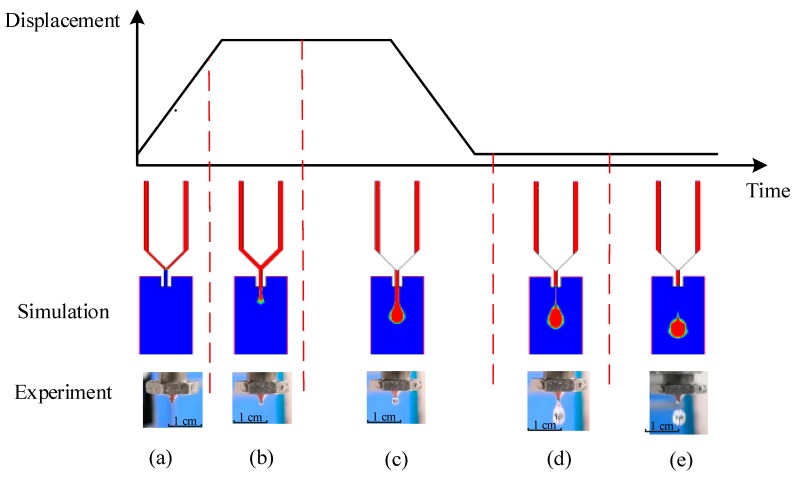
Morphological changes in the droplet in a jetting cycle: (**a**) Initial stage; (**b**) growth stage; (**c**) extension stage; (**d**) breakage stage; (**e**) separation stage.

For the needle-type piezoelectric jetting dispenser, a trapezoidal voltage signal was chosen to drive the piezoelectric actuator. Figure 4 shows the morphological changes in a droplet during the needle vibration process, where the droplet formation and separation process is divided into five stages. The first stage is the initial stage. As Figure 3b shows, the needle sat in the nozzle and blocked the adhesive in the chamber of the dispenser at the beginning. Then, driven by the piezoelectric actuator, the needle moved upward. The generated negative pressure between the nozzle and the needle made the liquid and air flow back into the nozzle as shown in Figure 4a. The second stage is the droplet growth stage. As Figure 4b shows, as the needle continued to move upward, the adhesive began to move along the channel and flow out from the nozzle driven by the air pressure in the liquid reservoir. The third stage is the droplet extension stage. An increasing amount of liquid flowed out from the nozzle in a long fluid stream format. The lower part of the stream showed a droplet shape. When the needle moved down and blocked the channel again, the stream reached its largest size as shown in Figure 4c. The fourth stage is the droplet breakage stage. As the needle had blocked the nozzle, no more liquid was able to flow out. However, the stream still moved downward due to inertia, which produced a reduced diameter point under the nozzle. At this point, the stream radius became smaller and eventually broke as shown in Figure 4d. The fifth stage is the droplet separation stage. The breakage separated the stream into two parts. The tapered tail of the ejected part flowed quickly to the main body because of surface tension, which produced a ball-shaped droplet. The other part under the nozzle was inhaled back during the next jetting cycle as shown in Figure 4e.

**Figure 5:**
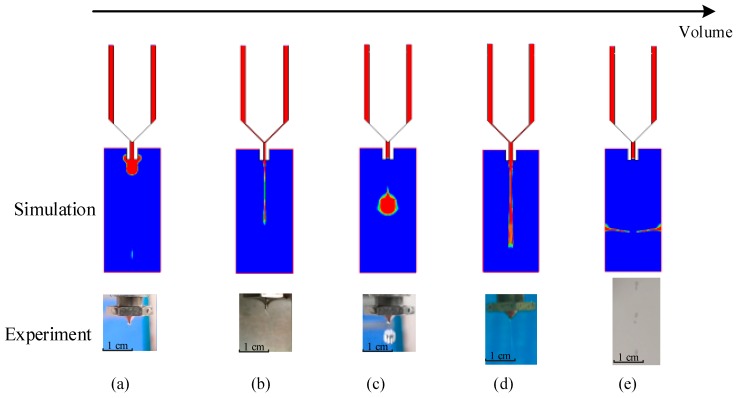
Droplet morphologies with different volumes: (**a**) Adhesion; (**b**) residual liquid; (**c**) normal droplet; (**d**) flow-stream; (**e**) scattered dots.

It can be seen from Figure 4 that a normal droplet with a desired volume can be obtained during a successful jetting cycle. However, if the system parameters do not fall into suitable ranges, some undesirable jetting failures will appear. For example, during the droplet growth stage, if the dispensed volume is too small, no matter the increase in the driving force, it is impossible to break the adhesive through the thick liquid layer under the nozzle (Figure 5a). When the volume is slightly increased, the liquid may form a droplet, but the amount of liquid that remains at the end of the nozzle will be too large, which reduces the dispensing accuracy (Figure 5b). On the other hand, if the dispensing volume is too large, it is particularly easy for the large amount of adhesive hanging underneath the nozzle to form a long liquid stream (Figure 5d); even worse, the stream will be separated into lots of scattered dots, as shown in Figure 5e.

The abovementioned jetting failures are related to the system parameter settings. In the next step, we further studied the effects of driving and structural parameters, including needle displacement, air pressure, viscosity, nozzle length, and diameter, on the morphological changes in the droplet to guide the control of its volume in production.

## 4. Influences of System Parameters on Droplet Volume and Morphology

**Figure 6:**
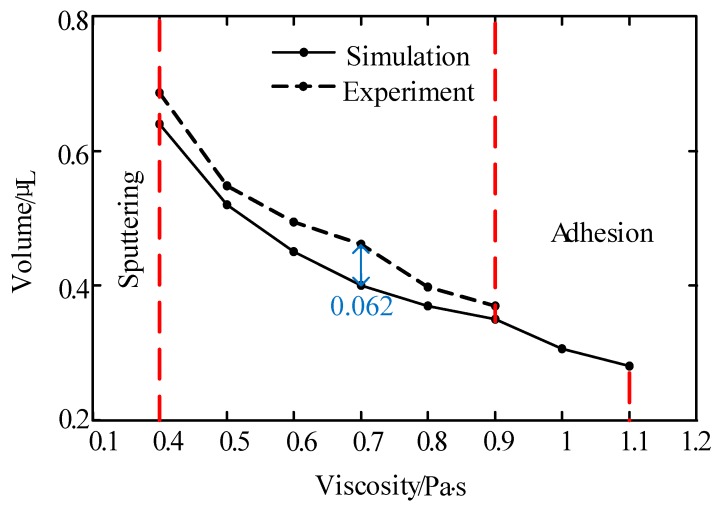
The influence of viscosity on the volume of the droplet. Conditions: The nozzle diameter is 0.2 mm, the needle stroke is 0.3 mm, the frequency is 20 Hz, and the pressure is 0.6 MPa.

**Figure 7:**
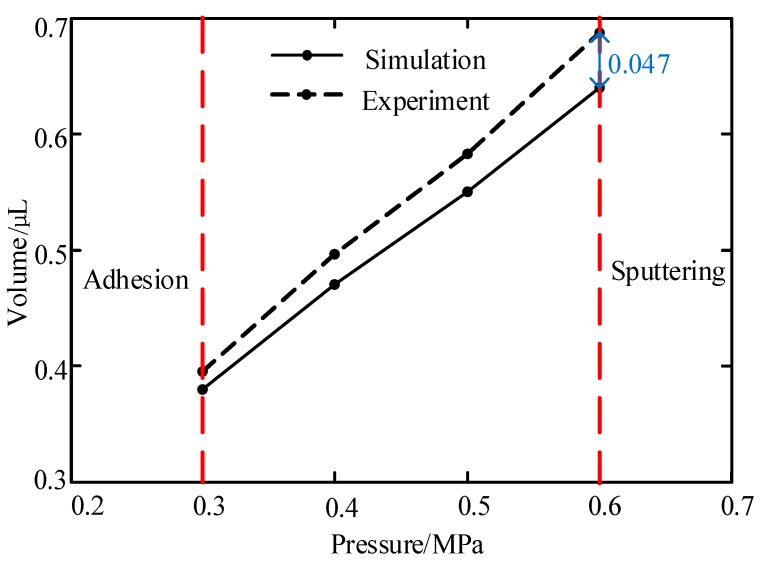
The influence of pressure on the volume of the droplet. Conditions: The nozzle diameter is 0.2 mm, the needle stroke is 0.3 mm, the frequency is 20 Hz, and the viscosity is 0.4 Pa·s.

**Figure 8:**
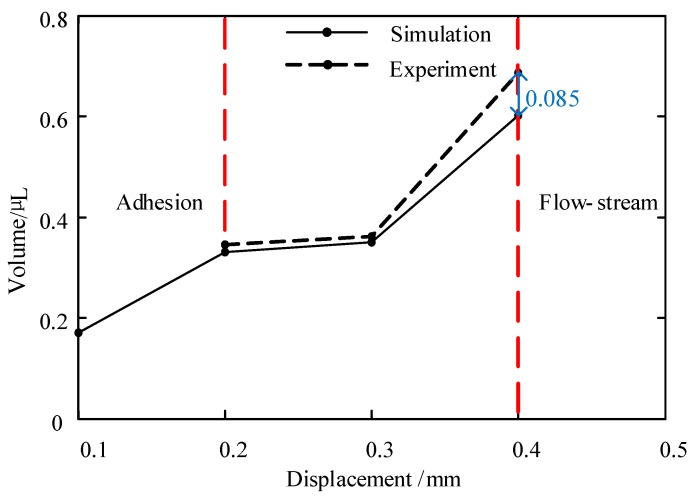
The influence of needle displacement on the volume of the droplet. Conditions: The nozzle diameter is 0.2 mm, the frequency is 20 Hz, the viscosity is 0.8 Pa·s, and the pressure is 0.6 MPa.

In this part, we conducted many experiments and simulations to study the influences. During the experiment, glycerol was adopted as the dispensing adhesive, whose viscosity was controlled by using the mentioned ceramic heating element. In order to reduce the impact of measurement error, we used the average of 10 experiments as the effective volume for each test, as shown in Figure 6, Figure 7 and Figure 8. And the maximum deviations of experiments and simulations are also displayed in the figures. 

First, we studied the influences of pressure and viscosity on droplet volume and morphology.

As Figure 6 shows, under the described jetting condition, the volume of the droplet decreases as the liquid viscosity increases. When the viscosity was lower than 0.4 Pa·s, the droplet separated into many dots or sputtered out. However, the flows could not be cut off when the viscosity was greater than 0.9 Pa·s during the experiment. It can be seen from Figure 7 that the increase in pressure enlarged the droplet size. When the pressure was less than 0.2 MPa, the system was unable to provide enough energy to drive the liquid to eject out from the nozzle, as shown in Figure 5b. On the other hand, when the pressure was greater than 0.4 MPa, the liquid separated into many satellite dots due to the excessive kinetic energy that was provided by the pressure.

**Figure 9:**
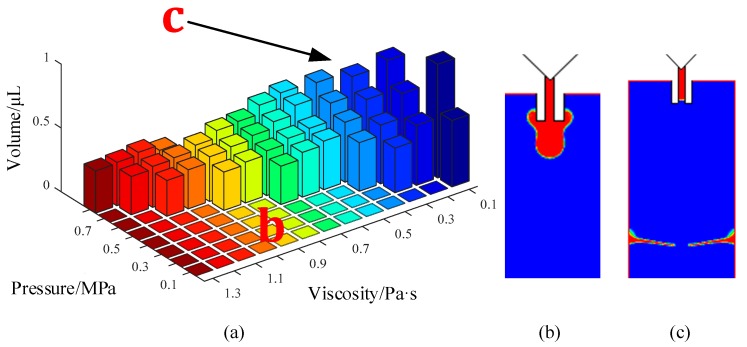
The comprehensive influence of pressure and viscosity on the droplet volume and morphology: (**a**) Jetting volume; (**b**) adhesion phenomenon; (**c**) satellite dots. Conditions: The nozzle diameter is 0.2 mm, the needle stroke is 0.3 mm, the frequency is 20 Hz, and the surface tension is 0.03 N/m.

Figure 9 shows the comprehensive influence of viscosity and pressure on droplet volume. The right part of Figure 9a shows that the volume values were set to zero due to the failure of the dispensing process. As the figure demonstrates, with an increase in pressure and a decrease in viscosity, a larger droplet can be ejected out from the nozzle. For the low-viscosity liquids, the droplet was formed under a smaller driving pressure. When the viscosity increases, the required pressure must be increased to overcome the restriction of the viscous force, otherwise the adhesive will hang on the nozzle tip and form a thick liquid layer (Figure 9b). Besides this, the volume of the droplet became smaller with the increase in viscosity under the same driving pressure. On the other hand, a large driving pressure is suitable for a high-viscosity adhesive. If the viscosity is too low, the excessive driving force will separate the droplet into many satellite dots (Figure 9c). Thus, the driving pressure should be controlled in the matching range to form a normal droplet with the required volume.

**Figure 10:**
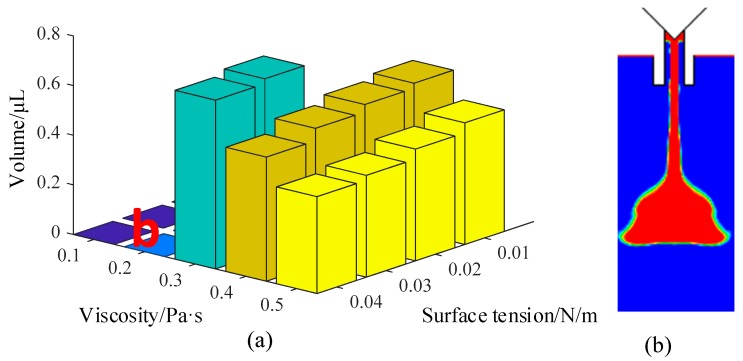
The influence of surface tension and viscosity on the volume of the droplet: (**a**) Jetting volume; (**b**) sputtering. Conditions: The nozzle diameter is 0.2 mm, the frequency is 20 Hz, and the needle displacement is 0.3 mm.

**Figure 11:**
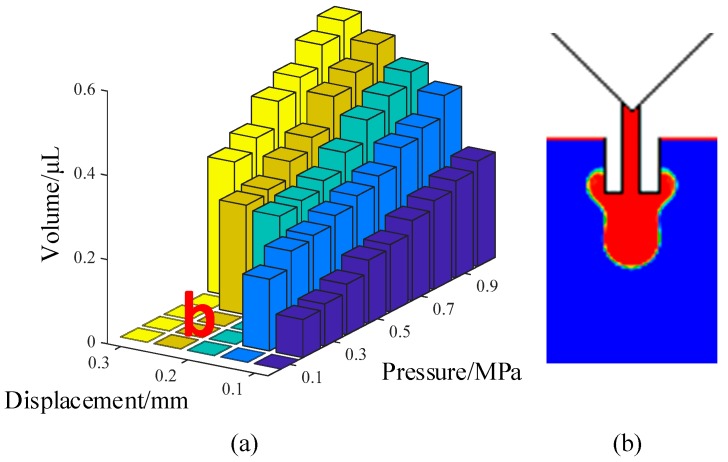
The influence of displacement and pressure on droplet volume and morphology: (**a**) Jetting volume; (**b**) adhesion. Conditions: The nozzle diameter is 0.2 mm, the viscosity is 0.8 Pa·s, the frequency is 20 Hz, and the surface tension is 0.06 N/m.

**Figure 12:**
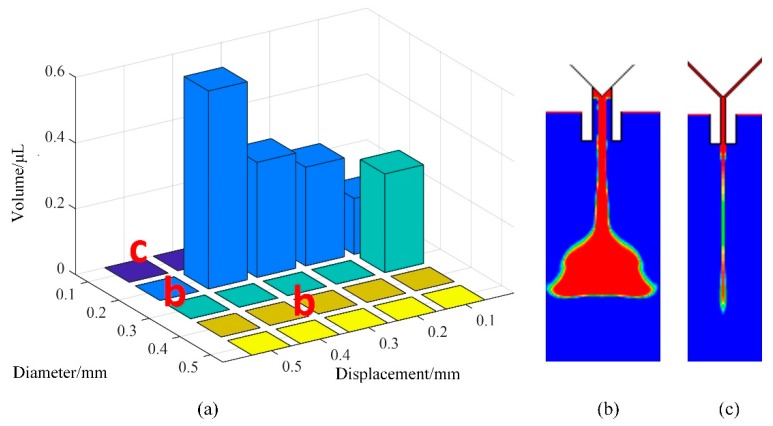
The influence of needle travel and nozzle diameter on droplet volume and morphology: (**a**) Jetting volume; (**b**) sputtering; (**c**) residual liquid. Conditions: The driving pressure is 0.6 MPa, the viscosity is 0.8 Pa·s, the frequency is 20 Hz, and the surface tension is 0.06 N/m.

In the next step, the influence of needle stroke on the droplet volume is studied. As Figure 8 shows, the droplet’s volume became larger with the increase in needle displacement. There are two main reasons for this phenomenon. One is that more liquid will flow out from the nozzle when the channel gap becomes larger, which is caused by the increase in needle displacement in a jetting cycle. The other reason is that the hitting force will become stronger due to the increase in the speed at which the needle falls. It can also be observed from the figure that a liquid adhesion phenomenon occurred when the displacement was lower than 0.2 mm during the experiment. This is caused by the small amount of liquid and the low driving force. On the other hand, when the displacement was larger than 0.4 mm, the excess of liquid formed a flow-stream but not a droplet. In this situation, the other parameters, such as air pressure and nozzle length, should be adjusted to balance the driving force. Figure 10, Figure 11 and Figure 12 show the comprehensive influences of the system parameters on the droplet volume.

Figure 10 shows the comprehensive influences of surface tension and viscosity on the droplet volume and formation process. As the figure shows, the droplets become smaller and smaller with the increase in surface tension. When the surface tension and the viscosity are both too small, the liquid easily flows out from the nozzle. As Figure 10b shows, the ejected liquid is stretched very long after the needle blocks the flow channel. These facts are caused by the mutual attraction of liquid molecules. When the surface tension is low, the attraction of the inner molecules of droplets to the surface molecules is very weak, which causes the droplet to appear long in shape. With the increase in surface tension, the agglomeration is significantly enhanced. The surface molecules are close to each other under the strong attraction of the inner layer molecules, and this makes the droplet have a ball shape. The simulations also show that a larger driving force is required with the increase of surface tension. 

Figure 11 shows the comprehensive effects of needle travel and driving pressure on the droplet volume and formation process. As depicted in this figure, the volume of the droplet was increased with the increase in needle displacement and driving pressure. When the pressure was within 0.2 MPa and the needle travel was within 0.2 mm, the system was unable to provide enough energy to drive the liquid to move out from the nozzle (Figure 11b). With the increase in needle displacement, more liquid moved out and hung at the nozzle’s tip. Even worse, the continuous increase in the needle stroke caused the air to flow into the nozzle during the needle-raising process. The adhesion and backflow situations are both caused by the lack of driving force. In these circumstances, the adhesive can be pushed out and separated into droplets by increasing the pressure. However, the pressure should be controlled within a suitable range to avoid a long stream.

In the next step, we studied the influences of needle travel and nozzle diameter on the volume and shape of the droplet. It can be seen from Figure 12 that the values of the parameters should also be controlled within suitable ranges to avoid jetting failures. When the needle travel parameter had a small value, we were able to obtain small droplets. With the increase in the displacement, the volume of the droplet became larger. When the displacement was greater than 0.5 mm, too much liquid flowed out from the nozzle in a jetting cycle, which led to the adhesion or flow-stream phenomenon (Figure 12b). Therefore, we recommend that the range of needle displacement be 0.1–0.4 mm to form a normal droplet. Moreover, as the figure shows, if the needle diameter is smaller than 0.2 mm, no matter how large the displacement is, the liquid will just hang underneath the nozzle’s tip or produce residual liquid (Figure 12c). This is because only a small amount of liquid flows out from the nozzle under the air pressure setting. With the increase in diameter, more liquid will flow out from the nozzle to form a larger droplet. When the diameter is too large, a long flow-stream will form under the nozzle, as shown in the right part of Figure 12a. Thus, it is important to choose a suitable nozzle for the adhesive to form a droplet with the required volume.

In addition, Figure 4, Figure 5, Figure 6, Figure 7, Figure 8 and Figure 9 list and compare the simulation and experimental results. It can be seen that the results match well in these figures, which demonstrates the validity and correctness of the simulation models.

## 5. Further Discussion

Based on the simulations and experiments, we provide the recommended ranges of parameter values and the desired droplet volumes for liquids with different viscosities in Table 2. These settings can help to ensure that the droplet is ejected out with the required volume and jetting speed on the premise of a successful formation and separation process. Besides this, it should be noted that some heating measures are required to reduce the viscosity of high-viscosity liquids to a suitable range.

## 6. Conclusions

We studied the influences of system parameters on droplet size for a needle-type piezoelectric jetting dispenser by considering a successfully-performed droplet ejection process through a numerical simulation. The configuration and working principle of the dispenser and the experimental platform were introduced before the simulation model was constructed. Then, the droplet formation and separation process and the morphological changes in the droplet with an increase in volume were studied by the finite volume method. The influences of system parameters, including air pressure, nozzle size, needle strokes, and liquid properties, on the droplet volume and morphology were comprehensively analyzed. The simulation and experiment results show that the droplet volume became larger with the increase in air pressure, needle stroke, and nozzle size until a flow-stream or satellite droplets appeared. On the other hand, a very small value for these parameters was found to lead to adhesion or micro-dots. For a high-viscosity liquid, one should choose a large nozzle and needle displacement to produce normal droplets. Finally, based on the simulation and experiment results, we also provided recommended ranges of parameter values and suitable droplet volumes for liquids with different viscosities, which can be used to guide the droplet volume control process for needle-type jetting dispensers. In a further study, we will undertake research on intelligent control of the dispensing process.

## Figures and Tables

**Table 1:** Ranges of simulation parameter values.

Viscosity (Pa·s)	Pressure (MPa)	Nozzle Diameter (mm)	Needle Stroke (mm)	Needle Frequency (Hz)	Density (kg/m^3^)	Surface Tension (N/m)
0.1–2	0.1–0.8	0.1–0.5	0.1–0.5	0–100	2	0.01–0.1

**Table 2:** The recommended parameter value ranges for liquids with different viscosities.

Viscosity (Pa·s)	Nozzle Diameter (mm)	Needle Stroke (mm)	Jetting Frequency (Hz)	Pressure (MPa)	Droplet Volume (μL)
<1.5	0.1–0.2	0.1–0.4	<250	<0.6	>0.01
1.5–2.5	0.1–0.3	0.2–0.5	<200	0.1–0.6	>0.04
2.5–3.5	0.2–0.3	0.2–0.5	<150	0.2–0.6	>0.1
3.5–5	0.2–0.5	0.3–0.6	<100	0.3–0.8	>0.2
